# Validation of Claims Data for Absorbing Pads as a Measure for Urinary Incontinence after Radical Prostatectomy, a National Cross-Sectional Analysis

**DOI:** 10.3390/cancers15245740

**Published:** 2023-12-07

**Authors:** Diederik J. H. Baas, Jan Reitsma, Lieke van Gerwen, Jaron Vleghaar, Jolanda M. L. G. Gehlen, Cathelijne M. P. Ziedses des Plantes, Jean Paul A. van Basten, Roderick C. N. van den Bergh, H. Max Bruins, Eelco R. P. Collette, Robert J. Hoekstra, Ben C. Knipscheer, Pim J. van Leeuwen, Daphne Luijendijk-de Bruin, Joep G. H. van Roermund, J. P. Michiel Sedelaar, Tommy G. W. Speel, Saskia P. Stomps, Carl J. Wijburg, Rob P. W. F. Wijn, Igle Jan de Jong, Diederik M. Somford

**Affiliations:** 1 Department of Urology, Canisius Wilhelmina Hospital, 6532 SZ Nijmegen, The Netherlands; 2 Prosper Prostate Cancer Clinics, 6532 SZ Nijmegen, The Netherlands; 3 Zorgverzekeraars Nederland, 3700 AM Zeist, The Netherlands; 4 Vektis Intelligence, Vektis, 3700 AS Zeist, The Netherlands; 5 CZ Zorgverzekeringen, 5038 KE Tilburg, The Netherlands; 6 Zilveren Kruis, 3833 LB Leusden, The Netherlands; 7 Department of Urology, St. Antonius Hospital, 3435 CM Nieuwegein, The Netherlands; 8 Department of Urology, Zuyderland Medical Center, 6419 PC Heerlen, The Netherlands; 9 Department of Urology, Bravis Hospital, 4624 VT Bergen op Zoom, The Netherlands; 10 Department of Urology, Catharina Hospital Eindhoven, 5623 EJ Eindhoven, The Netherlands; 11 Department of Urology, Treant Zorggroep, 7824 AA Emmen, The Netherlands; 12 Department of Urology, Netherlands Cancer Institute, 1066 CX Amsterdam, The Netherlands; 13 Department of Urology, Martini Hospital, 9728 NT Groningen, The Netherlands; 14 Department of Urology, Maastricht University Medical Center+, 6229 HX Maastricht, The Netherlands; 15 Department of Urology, Radboud University Medical Center, 6525 GA Nijmegen, The Netherlands; 16 Department of Urology, Leeuwarden Medical Center, 8934 AD Leeuwarden, The Netherlands; 17 Department of Urology, Ziekenhuisgroep Twente, 7609 PP Almelo, The Netherlands; 18 Department of Urology, Rijnstate Hospital, 6815 AD Arnhem, The Netherlands; 19 Department of Urology, Jeroen Bosch Hospital, 5223 GZ Hertogenbosch, The Netherlands; 20 Department of Urology, University Medical Center Groningen, 9713 GZ Groningen, The Netherlands

**Keywords:** healthcare administrative claim, urinary incontinence, prostate cancer, prostatectomy, quality of life, patient reported outcome measures

## Abstract

**Simple Summary:**

Radical prostatectomy (RP) is a common treatment for prostate cancer but has a risk of side-effects. Urinary incontinence (UI) after RP ranges from 4 to 31%, depending on the method of reporting and definitions used. The aim of this study was to evaluate if healthcare insurance claims data of absorbing pads in the Netherlands provide a valid alternative in the measurement of post-prostatectomy UI (defined as the use of ≥1 pad(s) per day), compared to self-reported pad use. Claims data and self-reported use was available for 416 patients. According to the claims data, patients had a UI rate of 31%, compared to a self-reported UI rate of 45% one year after RP. The agreement between both measures was moderate. Claims data can be considered as a conservative quality indicator for UI after RP.

**Abstract:**

The use of healthcare insurance claims data for urinary incontinence (UI) pads has the potential to serve as an objective measure for assessing post-radical prostatectomy UI rates, but its validity for this purpose has not been established. The aim of this study is to correlate claims data with Patient Reported Outcome Measures (PROMs) for UI pad use. Patients who underwent RP in the Netherlands between September 2019 and February 2020 were included. Incontinence was defined as the daily use of ≥1 pad(s). Claims data for UI pads at 12–15 months after RP were extracted from a nationwide healthcare insurance database in the Netherlands. Participating hospitals provided PROMS data. In total, 1624 patients underwent RP. Corresponding data of 845 patients was provided by nine participating hospitals, of which 416 patients were matched with complete PROMs data. Claims data and PROMs showed 31% and 45% post-RP UI (≥1 pads). UI according to claims data compared with PROMs had a sensitivity of 62%, specificity of 96%, PPV of 92%, NPV of 75% and accuracy of 81%. The agreement between both methods was moderate (κ = 0.60). Claims data for pads moderately align with PROMs in assessing post-prostatectomy urinary incontinence and could be considered as a conservative quality indicator.

## 1. Introduction

Radical prostatectomy (RP) as a primary treatment for localized prostate cancer (PCa) bears a risk of relevant side-effects such as urinary incontinence (UI) and erectile dysfunction which impair quality of life (QoL) [1]. UI is often at its worst in the first few months after RP and improves up until 12 months. After that, a plateau is reached with only a slight improvement up to 24 months [2]. Prior studies reported UI rates ranging from 4 to 31% one year after RP [2,3,4]. As different definitions of UI and methods of registration were used, the comparability between these studies is low. 

Also, several patient-related factors have been identified that may contribute to post-RP UI such as age [5], BMI, prostate volume and urethral length [6,7,8], but surgeon’s experience and hospital RP volume might also be of influence [9,10,11,12,13]. 

To assess post-RP UI, several methods are available. Often, post-RP UI is evaluated with commonly used patient-reported outcome measures (PROMs) and can be indexed as a composite continence score from such validated questionnaires. Several PCa-specific and validated questionnaires are available to measure QoL among PCa patients. In the Netherlands, the Expanded Prostate Cancer Index Composite (EPIC-50), or short form version (EPIC-26), or the International Consultation on Incontinence Questionnaire-Short Form (ICIQ-SF) questionnaires are most frequently used [14,15,16]. As an alternative to composite scores, the number of absorbing pads used per day can be extracted from patient files following an interview in the outpatient department or registered as part of validated questionnaires as a single question on the number of pads used on a daily basis (e.g., question 3 of the EPIC 26 questionnaire: ‘How many pads or adult diapers per day did you usually use to control leakage during the last 4 weeks?’; with the following possible multiple choice answers: none/1 pad per day/2 pads per day/ 3 or more pads per day). 

When using the number of pads used per day, several definitions of urinary continence are reported in the literature. Most studies define continence as the use of no pads at all. Holze et al. compared the number of daily pads used with patients’ perceived continence [17]. They found that the use of no pads better reflects patients’ subjective assessment of continence, compared to the use of 0–1 pads, often referred to as ‘social continence’. The reported number of pads used is, however, subjective and could depend on patients’ hygienic preferences. Another method of assessing post-RP UI is the use of weighing pads and defining urinary loss in grams or ml per time unit. 

Evaluating post-RP UI can be challenging using PROMs. To evaluate QoL following RP, PROMs need to be integrated into clinical care, which takes effort and increases costs of the care chain, while its reliability as a general quality indicator depends heavily on the patient response rate. Prior studies have demonstrated that the response rate is often disappointing, and a lot of effort is required to increase this [18]. Therefore, alternative methods to assess UI rates for long-term monitoring and adequate comparison between hospitals are urgently needed. 

In the Netherlands, healthcare insurance is mandatory for all citizens. This offers the opportunity to analyze nationwide claims data within the centrally registered National Claims Database (Vektis) in which all insurance companies in the Netherlands take part. This approach has been successful in the past for quality assessment in myocardial infarction, chemotherapy and intensive care unit admissions in upper gastrointestinal cancer patients, as well as other diseases [19,20,21,22], resulting in the identification of new quality indicators. In 2018, Schepens et al. evaluated the post-RP UI rate based on the claims of absorbing pads [23]. They found that 26% of patients suffer from post-RP UI, defined as the usage of at least one pad per day one year postoperatively. Furthermore, they found significantly lower UI rates in hospitals in which > 100 procedures were performed yearly. Based on this and other international studies elaborating on the hospital volume–outcome relationship, the minimum RP volume per hospital in the Netherlands was raised from 20 procedures to 50 procedures annually in 2018 and up to 100 procedures in 2019.

Recently, the short-term effect of raising the minimum volume for RP procedures per hospital on post-RP UI in the Netherlands was re-evaluated using the same methods [24]. Despite raising the minimum volume per hospital to 100 per year, the post-RP UI rate on a national level did not significantly improve in the short-term. Also, a wide range of post-RP UI still persisted in both low- as well as high-volume hospitals. While it is very plausible that the effect of the installation of the minimum volume standard would need more time to have its maximum effect on post-RP UI rates, the authors concluded that other measures, such as measuring outcomes on a per-surgeon level and more focus on audit and feedback, are necessary in addition to raising the minimum volume to relevantly improve outcomes for patients. 

To introduce meaningful quality assurance programs for RP evaluating its outcomes, the availability of reliable and reproducible data for the majority of patients is of paramount importance for cyclic evaluation and claims data should be considered for this specific purpose. There is, however, one caveat when using claims data for absorbing pads as a quality indicator; the use of claims data has not been validated for this purpose. The aim of this study is to validate pad-usage based on claims data with PROMs data for post-RP UI.

## 2. Materials and Methods

Patients were retrospectively identified based on the declaration of an RP with their insurance company (ZA code 036556 and 036553) between 1 September 2019 and 29 February 2020 in the National Claims Database (Vektis). Patients were excluded from analysis if there was a history of pad claims 30–120 days prior to RP. Patients that underwent continence surgery (urethral sling, bulking agents or implementation of sphincter prosthesis) within 15 months after RP were excluded, as this could give a mismatch between the reported pad use and claimed pad use for the same period [25,26]. Patients that did not have healthcare insurance or died within 15 months after RP were also excluded from analysis. 

The claims of pads with healthcare insurance providers are carried out according to daily incontinence patterns, with corresponding profiles (Table 1), 12–15 months after RP. As declarations are registered at the end of the period in which they are used, this time frame was chosen to have the most accurate estimation of pad use around the 12 months post-RP mark. 

If a patient’s claim history was not registered using a profile, a corresponding profile was estimated based on the average monthly costs of the claimed pads. Patients with claims corresponding to profile 3 or higher were grouped. 

To evaluate the PROMs data, all hospitals in the Netherlands performing RP were asked to participate in this study. Nine out of seventeen hospitals agreed to participate in this study. In participating centers, ethical approval was obtained according to local protocols. To include patients who responded to PROMs around the one year follow-up visit, participating hospitals were requested to share the number of daily pads used for UI, 10–15 months after RP as reported with the validated EPIC-26 question 3 or EPIC-50 question 5 (“how many pads or adult diapers per day did you usually use to control leakage during the last 4 weeks?”) on a per patient level. Patients were excluded from the PROMs dataset if reported pad use according to PROMs was missing, or if the reported PROMs were collected outside of the 10–15 month timeframe. 

After pseudonymization by a trusted third party (Stichting ZorgTTP, Houten, the Netherlands), corresponding patients with valid PROMs data and claims data were matched on the combination of date of birth and date of surgery. 

To find the best possible concordance with pad use according to PROMs, continence according to claims data was defined as no claims or claims within profile 0. UI was defined as claims within profile 1 or higher (Table 1), corresponding with the use of at least one pad per 24 h. UI according to PROMs was defined as the reported usage of 1 or more pad per day. For patients with reported PROMs, the usage of ≥2 pads per day was also investigated, as both definitions for UI are used in the literature [2,3,27]. 

Sensitivity, specificity, positive predictive value (PPV), negative predictive value (NPV), and accuracy of claims data as measures for UI as reported with PROMs were calculated. The level of agreement between claims data and PROMs was measured with Cohen’s kappa coefficient [28]. All statistical tests were considered statistically significant at *p* values of <0.05 and were conducted with SPSS Statistics (IBM, version 27.0).

## 3. Results

### 3.1. Claims Data 

In the claims dataset, 1624 patients were identified. A total of 40 patients were excluded due to UI before RP (*n* = 21), death within 15 months after RP (*n* = 10), uninsured status (*n* = 5), or for continence repair surgery (*n* = 4).

In the claims dataset (*n* = 1584), the majority of patients (65%, Table 2) did not claim any pads for UI or did so according to profile 0 (4%) and were considered continent. 

Reimbursement of pads for UI was carried out according to one of the profiles in 71% of the patients that claimed pads. In the remaining 29% of patients, a corresponding profile was estimated based on the average costs of the claims made. The observed UI rate on patient level according to claims data was 31% (Table 2). There was a comparable UI rate between patients for whom PROMs were reported (*n* = 432) compared to those for whom PROMs were not reported (*n* = 1152); 31% vs. 30%, *p* = 0.739. The observed mean UI rate on the hospital level (unadjusted for volume) was 34% and varied importantly between the 17 hospitals from 10% to 64%.

### 3.2. PROMs Data and Validation

Nine out of seventeen hospitals performing RP in the Netherlands participated in this study and provided data for a total of 845 patients. Of these, patients were excluded in the case of missing PROMs data during the study period (*n* = 400), leaving 445 patients for the validation of claims data. Patients were divided into a continent or an incontinent group, based on their PROMs and claims data. Out of 445 patients, 416 patients from the PROMs data were successfully matched by the trusted third party with their corresponding record from the claims data. The use of ≥1 pad(s) per day was 45% according to PROMs data and 30% according to claims data. Usage of ≥2 pads according to PROMs data was 13%. Due to the nature of the claims profiles (Table 1), this could not be assessed for claims data as patients with an average claim of two pads can be placed into different profiles. Sensitivity was 62%, specificity 96%, PPV 92%, NPV 75% and accuracy 81% for incontinence (≥1 pad(s) per day) according to claimed pads and reported use of pads (Table 3). The level of agreement between PROMs data and claims data was moderate (κ = 0.60). 

## 4. Discussion

We found a moderate level of agreement between claims data of absorbing pads and PROMs to assess post-RP UI rates. Based on the PROMs data, long-term post-RP UI rates were 45% for ≥1 pad(s) and 13% for ≥2 pads. These rates are lower compared to a prior Dutch nationwide study conducted in 2015/2016, in which post-RP daily pad use at 12 months according to the EPIC-26 was 53% for ≥1 pad(s) and 21% for ≥2 pad(s) [2]. A systematic review and meta-analysis from 2012 evaluating urinary continence recovery after RP showed a varying UI rate at 12 months, ranging from 4% to 31% [3], with a mean UI rate (any pad use at 12 months) of 16%, which is less than the 45% UI rate in our study. However, the method of data collection (validated questionnaires, interviews, unclear or not reported) and the definition of continence are heterogenous, which complicates comparisons between studies.

The post-RP UI rate 12–15 months after RP based on claims data was 31% in this study. A similar study was performed in Austria and reported that for patients with minimal invasive RP, 24.7% received a pad prescription postoperatively, which remained stable during the follow-up period of 36 months [29]. However, their figures were based on the monthly rate of prescribed pads, which makes it hard to compare their results with our study. Tollefson et al. evaluated the correlation of ICD-9 billing codes with PROMs for UI pad use and found a poor correlation (kappa coefficient of 0.169 for any pad use) [30]. The authors conclude that registration data are inaccurate for usage. Their study was similar in design, but their validation was based on ICD-9 data codes with possible registration errors and consequently bias, which makes a direct comparison with our data difficult to interpret. 

The reported UI with PROMs was higher than might be expected based on the claims data (45% vs. 31%). Interestingly, of 189 patients that reported to use at least one pad per day with PROMs, 71 (38%) did not claim any incontinence pads. A possible explanation for this may be that patients with mild UI buy pads in a drugstore or dispensary. Perhaps some men are not aware of the possibility for reimbursement of these pads. Conversely, only 10 out of 128 patients (7.8%) had a history of pad claims but reported to be continent. 

These results show that claims data of absorbing pads provide a valid but conservative indicator for post-RP UI rates, compared to PROMs data. This is supported by the unlikeliness of patients claiming incontinence pads, while not reporting incontinence in their PROMs data (specificity 96%, PPV 92%). It is a conservative measure, since not all patients reporting incontinence in the PROMs data actually claimed incontinence pads (sensitivity was 62%, NPV 75%), which explains the moderate general agreement (accuracy 81%; κ = 0.60). The interpretation of incontinence rates based on claims data should take into account this conservative character of the data, since incontinence rates based on PROMs data are higher.

The strength of our study is that it represents a nationwide survey of claims data including most patients that underwent RP in the study period and that we used a strict definition of UI. Several limitations have to be recognized. The PROMs data consists of a quarter of the total population, as not all hospitals performing RP in the Netherlands participated in the study or are offering PROMs. Many PROMs data were missing due to low response rates. The observed UI rate of claims data was similar for patients with and without PROMs, which gives us confidence that the PROMs data are not biased with regard to the UI rate. 

Due to the retrospective nature of this study, there is a risk of selection bias and confounding when comparing the overall UI rates between centers. We did not adjust for factors influencing patients’ continence rates such as hospital volume, surgeon experience, disease characteristics or treatment modality (open, laparoscopic or robot-assisted) as this was a cross-sectional study, aimed to validate claims data with PROMs data and therefore not affecting the main outcome of our study. Further research is needed to investigate the impact of these factors on the widely varying UI rates between hospitals.

Lastly, we used pad use per 24 h as a measurement unit, which is a relative rough measure as pads come in different size and capacities, and the usage of pads can depend on hygienic preferences. Wallerstedt et al. investigated the different definitions of post prostatectomy UI and correlated the number of pads used with the bother experienced from urinary leakage [31]. They reported that even a small rate of urinary leakage can lead to clinically relevant bother. However, the more pads used, the greater the risk of bother experienced from urinary loss. Therefore, the actual grade of urinary leakage might not be the only relevant indicator. Several other studies elaborated on the correlation of pads used and the amount of urinary loss, with varying results. Tsui et al. reported that pads used and the severity of UI correlated weakly [32]. However, this was examined in incontinent patients without prior RP and in women, up to 95 years old, and might, therefore, not correspond with post-RP patients. Similar results were reported by Sacco et al. when evaluating the 48 h pad count and pad weight in a group of almost 15,000 incontinent patients, of which the mean age was 81 years [33]. Conversely, when evaluating pad use in post-RP patients, Nitti et al. reported perception of pads used and actual used pads in a post-RP cohort and found an excellent concordance of the number of pads used. These findings suggest that the use of the number of pads used as a measure of UI is dependent on the population studied. 

Ideally, future research should evaluate urinary loss with an objective measure such as loss in volume (mL) per 24 h, in order to correct for the subjective use of pads per 24 h, as Pham et al. recently demonstrated that PROMs may be used as a substitute for weighted pads in the early post-RP period [34]. Although this is the most laborious method of measuring UI, validating claims data with weighing pads per 24 h might be the most precise method of validating claims data.

Several studies evaluated epidemiological factors of PCa and outcomes after RP with administrative datasets, such as Medicare and the Surveillance, Epidemiology and End Results (SEER) program [35]. As noted by Penson et al. these administrative datasets offer valuable information regarding outcomes after RP but were not designed as quality indicators, which must be kept in mind when interpreting results from such studies. This applies to the National Claims Database as well, as it was not designed for this purpose. Our validation study showed moderate agreement, with high specificity and PPV. The wide variation in UI rates between hospitals calls for action to improve the outcomes for patients. Therefore, we suggest that Dutch claims data should be considered as a valid but conservative quality indicator to monitor UI rates over time and between hospitals. Whether this also applies to other countries depends on a nation’s healthcare and reimbursement system. It is recommended when using administrative data sets as quality indicator to validate these datasets with established reference standards such as PROMs. 

## 5. Conclusions

Claims data for absorbing pads show a moderate agreement with PROMs data for post-prostatectomy urinary incontinence and could be considered as a conservative quality indicator when differentiating between continent and incontinent patients.

## Figures and Tables

**Table 1:** Profiles of claims according to urinary incontinence pattern (source: Vektis).

Profile	Urinary Incontinence Pattern	Pads Used per Day
No claims	-	-
Profile 0	Low frequent, mild loss	<1 pads
Profile 1	<100 cc per 24 h	1–2 pads
Profile 2	<300 cc per 24 h	1–3 pads
Profile 3	<900 cc per 24 h	2–3 pads
Profile 4	<1500 cc per 24 h	3 pads
Profile 5	>1500 cc per 24 h	2–4 pads
Profile 6	>2000 cc per 24 h	3–5 pads

**Table 2:** Frequencies of pad claims according to healthcare insurance profiles.

Profile	*n* (%)
No claims	1038 (65%)
Profile 0	61 (4%)
Profile 1	16 (1%)
Profile 2	93 (6%)
Profile 3 or higher	376 (24%)
Total	1584 (100%)

**Table 3:** A 2 × 2 table of urine incontinence according to claims data and PROMs data.

		PROMs Data			Total
		0 Pads	1 Pad	≥2 Pads	
Claims data	No claims or profile 0	217	60	11	288
	Profile ≥ 1	10	72	46	128
Total		227	132	57	416

## Data Availability

Data are contained within the article.
